# Alterations in NO- and PGI_2_- dependent function in aorta in the orthotopic murine model of metastatic 4T1 breast cancer: relationship with pulmonary endothelial dysfunction and systemic inflammation

**DOI:** 10.1186/s12885-018-4445-z

**Published:** 2018-05-22

**Authors:** E. Buczek, A. Denslow, L. Mateuszuk, B. Proniewski, T. Wojcik, B. Sitek, A. Fedorowicz, A. Jasztal, E. Kus, A. Chmura- Skirlinska, R. Gurbiel, J. Wietrzyk, S. Chlopicki

**Affiliations:** 1 Jagiellonian Centre for Experimental Therapeutics (JCET), Jagiellonian University, Bobrzynskiego 14, 30-348 Krakow, Poland; 2 Department of Experimental Oncology, Hirszfeld Institute of Immunology and Experimental Therapy, Polish Academy of Sciences, R. Weigla St. 12, 53-114 Wroclaw, Poland; 3 Department of Molecular Biophysics, Faculty of Biotechnology, Jagiellonian University, Gronostajowa 7, 30-387 Krakow, Poland; 4 Chair of Pharmacology, Jagiellonian University, Medical College, Grzegorzecka 16, PL 31-531 Krakow, Poland

**Keywords:** Metastasis, Endothelial dysfunction, Inflammation, 4T1

## Abstract

**Background:**

Patients with cancer develop endothelial dysfunction and subsequently display a higher risk of cardiovascular events. The aim of the present work was to examine changes in nitric oxide (NO)- and prostacyclin (PGI_2_)-dependent endothelial function in the systemic conduit artery (aorta), in relation to the formation of lung metastases and to local and systemic inflammation in a murine orthotopic model of metastatic breast cancer.

**Methods:**

BALB/c female mice were orthotopically inoculated with 4T1 breast cancer cells. Development of lung metastases, lung inflammation, changes in blood count, systemic inflammatory response (e.g. SAA, SAP and IL-6), as well as changes in NO- and PGI_2_-dependent endothelial function in the aorta, were examined 2, 4, 5 and 6 weeks following cancer cell transplantation.

**Results:**

As early as 2 weeks following transplantation of breast cancer cells, in the early metastatic stage, lungs displayed histopathological signs of inflammation, NO production was impaired and nitrosylhemoglobin concentration in plasma was decreased. After 4 to 6 weeks, along with metastatic development, progressive leukocytosis and systemic inflammation (as seen through increased SAA, SAP, haptoglobin and IL-6 plasma concentrations) were observed. Six weeks following cancer cell inoculation, but not earlier, endothelial dysfunction in aorta was detected; this involved a decrease in basal NO production and a decrease in NO-dependent vasodilatation, that was associated with a compensatory increase in cyclooxygenase-2 (COX-2)- derived PGI_2_ production.

**Conclusions:**

In 4 T1 metastatic breast cancer in mice early pulmonary metastasis was correlated with lung inflammation, with an early decrease in pulmonary as well as systemic NO availability. Late metastasis was associated with robust, cancer-related, systemic inflammation and impairment of NO-dependent endothelial function in the aorta that was associated with compensatory upregulation of the COX-2-derived PGI_2_ pathway.

## Background

The vascular endothelium, considered the body’s largest autocrine/paracrine/endocrine organ, responds to various physical and chemical stimuli and maintains vascular homeostasis. Endothelial dysfunction, characterized by impaired production of vasoprotective endothelial mediators such as nitrite oxide (NO) and excessive activity of pro-oxidant, pro-thrombotic, pro-inflammatory mediators, plays a key role in the development of atherosclerosis and its cardiovascular complications, as well as in other cardiovascular diseases [1–3]. Cardiovascular complications observed in cancer patients and cancer survivors [4, 5] are also associated with the development of endothelial dysfunction that displays a similar phenotype to that described in patients with *atherothrombosis* [6].

In target organs for metastatic spread, endothelial inflammation may promote cancer metastasis. The endothelium acts as a barrier to cancer cell migration from blood to tissue. It has been proposed that the migration and invasion of circulating cancer cells into distant tissues is supported by the same mechanisms as those involved in leukocyte recruitment [7]. In fact, tumour cells appear to respond to the chemokines and cell adhesion molecules engaged in leukocyte trafficking and endothelial inflammatory response [8, 9]. Interestingly, recent in vitro studies have demonstrated that factors released from dysfunctional endothelium activate NF-κB and STAT3 signalling pathways within cancer cells, and promote their invasiveness in vitro [10]. At the same time, vasoprotective endothelial mediators such as for example prostacyclin (PGI_2_) display anti-metastatic activity, further supporting a crucial relationship between endothelial function and cancer metastasis [11, 12].

Metastases development is therefore related with changes of the endothelial phenotype in the affected organs (e.g. lung, liver) that contribute to the pathophysiology of metastasis. In addition, development of systemic endothelial dysfunction (e.g. in the conduit arteries) may contribute to cardiovascular complications in cancer patients. Obviously, the mechanisms mediating local changes in endothelial phenotype at metastatic sites and those involved in the development of systemic endothelial dysfunction may be different.

Among various available mouse models of cancer metastasis, the most appropriate for studying local and systemic endothelial response would be models, in which cancer cells are transplanted into the tissue of their origin (e.g. mammary gland, prostate or intestine), so that they form tumours that develop and progress from primary lesions to metastases in their natural microenvironment, i.e. as orthotopically-inoculated cancer cells syngrafted into immune-competent mice.

In the present study, we took advantage of the spontaneously metastasizing 4T1 murine mammary gland carcinoma model to examine the alterations in endothelial NO- and PGI_2_-dependent function in aorta that accompany metastatic expansion. The prolonged disease progression in a model established in our laboratory allowed us to identify a temporal relationships between the development of local, pulmonary inflammation, alterations in pulmonary endothelial function, systemic inflammation and endothelial dysfunction in conduit vessel (aorta) in relation to primary tumour growth and lung metastases. We showed that an early decrease in pulmonary and systemic NO availability coincided with the development of lung inflammation in the early metastatic phase, while late systemic endothelial dysfunction in aorta coincided with robust, systemic, cancer-related inflammation.

## Methods

### Animals

Seven- to eight-week-old BALB/c female mice were obtained from Charles River Laboratories Polska -Animal Lab (Poznan, Poland). Mice were housed in specific pathogen-free conditions (SPF) and fed a standard laboratory diet and water ad libitum.

All experimental procedures used in the present study were followed according to the Guidelines for Animal Care and Treatment of the European Communities and the Guide for the Care and Use of Laboratory Animals published by the US National Institutes of Health (NIH Publication No. 85–23, revised 1996). All procedures were approved by the First Local Ethical Committee on Animal Testing at the Jagiellonian University (Krakow, Poland), permit no: 140/2013.

### Tumour cell line

Mouse mammary adenocarcinoma 4 T1 cells were obtained from American Type Culture Collection (ATCC). Cells were cultured in RPMI 1640 (Laboratory of Analytical Chemistry, IIET) with Opti-MEM® media (Life Technologies) (1:1 *v*/v) and 5% fetal bovine serum (HyClone, Thermoscientific), supplemented with 4.5 g/L glucose, 2 mM glutamine, 1.0 mM sodium pyruvate (all from Sigma-Aldrich) and antibiotics (penicillin and streptomycin; Polfa Tarchomin). Cell cultures were maintained at 37 °C in a humidified atmosphere with 5% CO_2_.

### Murine model of metastatic breast cancer

1 × 10^4^ viable 4 T1 tumour cells suspended in 0.05 ml of Hanks Balanced Salt Solution were orthotopically inoculated into the right mammary fat pad of female BALB/c mice. Analyses were conducted at 2, 4, 5 and 6 weeks after 4 T1 cancer cell transplantation. Prior to each analysis, animals were randomly divided into two experimental groups (one group designated for the analysis and the other for further tumour development), so that the mean tumour volumes and tumour volume distributions were similar between experimental groups. Healthy BALB/c mice were used as a control group, and were analysed simultaneously with the tumour-bearing mice at 2, 4, 5 and 6 weeks after 4 T1 cancer cell transplantation.

Animals were anesthetized by intraperitoneal (*i.p*.) injection of a mixture of ketamine and xylazine (100 mg ketamine and 10 mg xylazine/kg body weight). Blood samples were collected from the right ventricle of the heart using a syringe containing anticoagulant (EDTA 1.6 mg/ml).

### Assessment of the primary tumour and number of metastases in the lungs

Primary tumours were carefully dissected from the surrounding tissues and weighed. Isolated lungs were washed in saline, weighed, and fixed with 4% formalin buffered solution. The number of metastases was macroscopically assessed; metastatic sites visible on the lung surface were visually counted using a magnification glass.

Macroscopic analysis of the lungs was followed by paraffin embedding, histological HE staining and histopathological assessment of the tissue.

### Histological and immunohistochemical analysis of the lungs

Lungs were fixed in 4% buffered formalin (for at least 48 h). After macroscopic analysis of the number of metastases, the lungs were prepared using the paraffin method, cut into 6 μm sections on an Accu-Cut® SRM™ 200 Rotary Microtome and stained with hematoxylin and eosin.

Light microscopic examination and photographic documentation were performed using an Olympus BX53F microscope equipped with a digital camera. Pictures were taken under the magnification 20× and 200×.

For immunohistochemical staining of VCAM-1 and vWF in the lung vasculature following deparaffinization, sections were pretreated according to the citrate-based HIER protocol and then preincubated with 5% goat serum (Jackson ImmunoResearch) and 2% dry milk to minimalize non- specific binding of antibodies. Primary rat-anti-mouse VCAM-1 (Chemicon) or rabbit-anti-mouse vWF (Abcam) antibodies were used, followed by Cy3-conjugated goat-anti-rat or Cy3- conjugated goat-anti-rabbit secondary antibodies (Jackson ImmunoResearch), respectively. Images were acquired using the AxioObserver D2 inverted fluorescent microscope (Carl Zeiss) and an AxioCam HRm monochromatic digital camera and stored as TIFF files. Fluorescence intensity was analysed automatically by Columbus software (Perkin Elmer).

### Assessment of NO production in the isolated lung preparation

Lungs were isolated from anaesthetized (pentobarbitone, 140 mg/kg, *i.p.*) animals and perfused at a constant flow of about 1.50 ml/min with low glucose DMEM with 4% albumin and 0.3% HEPES, mounted in a water-jacketed artificial thorax and ventilated with negative pressure at a rate of 90 breaths/min. The end-expiratory pressure in the chamber was set to − 3 cm H_2_O and inspiratory pressure was adjusted between − 6 and − 10 cm H_2_O to yield an initial tidal volume (TV) of about 0.2 ml. Every 5 min during the experiment, a deep breath of − 21 cm H_2_O end-inspiratory pressure was automatically initiated in order to avoid atelectasis. The PAP was set to around 3 cm H_2_O. Venous pressure was set to 2–5 cm H_2_O. All lungs preparations were allowed to equilibrate for at least 15 min under perfusion. Nitrate and nitrite concentrations were then measured in the effluent from the isolated lungs perfused with constant, non-recirculating flow. The samples were analysed via sensitive high-pressure liquid chromatography (HPLC) –based techniques (ENO-20 NOx Analyser; EiCom, Kyoto, Japan).

### Assessment of NO-dependent endothelial function in isolated rings of mice aorta

Mice aorta preparations and endothelial function assessment of aortic rings were conducted as previously described [13]. Briefly, NO-dependent endothelial function was measured by response to acetylcholine (Ach; 0.01–10 μM) in phenylephrine (Phe; 0.1–1 μM) pre-contracted vessels. Endothelium-independent vasodilatation was determined using sodium nitroprusside (SNP; 0.001–1 μM). To ensure that entire Ach-evoked response was NO-dependent the response to Ach and SNP was also measured in the presence of L-NAME. Responses were recorded using a data acquisition system and recording software (Power Lab, Lab Chart, AD Instruments, Australia).

### Assessment of nitrite production in isolated aorta rings

Basal NO production by the aorta was estimated using measurements of nitrite. Segments from the aortic arch were longitudinally opened, placed in a 96-well plate with endothelium facing up, and incubated for 1 h in 120 μl K-H buffer at 37 °C using a specially-designed closed chamber (BIO-V (Noxygen)) that was equilibrated with carbogen gas mixture (95% O_2_, 5% CO_2_). Samples from the incubation buffer were put on ice and used for measurement of nitrite with reductive gas-phase chemiluminescence in 1% wt/vol KI in acetic acid using Sievers* Nitric Oxide Analyzer NOA 280i, according to the manufacturer’s instructions. The averaged blank signal (without aortic rings) for a given set of experiments was subtracted as a background signal, to account for nitrite contamination in the buffer and/or laboratory atmosphere. Nitrite concentration was expressed as ng/ml/mg of dry weight of aortic rings.

### Assessment of PGI_2_ production in isolated rings of mice aorta

PGI_2_ production by aortic rings was quantified on the basis of the formation of 6-keto PGF1_α_, a stable metabolite of PGI_2_, in the supernatant of the aortic rings.

Aortic rings were incubated on a thermoblock (Liebisch Labortechnik) at 37 °C, in 1 ml of K-H buffer equilibrated with carbogen gas mixture (95% O_2_, 5% CO_2_), either in the absence or presence of selective COX-2 inhibitor DuP-697 (1 μM) or nonselective COX-1/COX-2 inhibitor indomethacin (5 μM). Both DuP-697 and indomethacin were dissolved in DMSO. Control rings were incubated with DMSO (1 μl/ml).

After equilibration and pre-incubation in the presence or absence of inhibitors, the aortic rings were placed in fresh buffer and incubated with or without inhibitors for 30 min. Effluent samples were taken after 3 (initial) and 30 (final) minutes of incubation. PGI_2_ production was calculated as the difference between final and initial 6-keto PGF1_α_ concentrations and was expressed as pg/ml/mg of dry weight of aortic rings. 6-keto-PGF-1_α_, concentration was measured using an EIA kit (Enzo, Life Technologies).

### Immunohistochemistry of aortic endothelium

Aortic rings were placed perpendicularly in OCT compound (Thermo) and then snap-frozen at − 80 °C. The blocks were mounted on the cryostat holder and cut into 10-μm-thick cross-sectional slides using the Leica CM1950 automatic cryostat. The sections were placed on polylisine-covered (Sigma-Aldrich) microscopic slides (Super Frost, Mentzel Gläser), and then acetone fixed (10 min). Pre-incubation with 2.5% horse serum (Vector Labs) and 2% dry milk was performed to minimize non-specific binding of antibodies. For indirect immunohistochemical detection of von Willebrand factor in endothelium, sections were incubated inside humid chambers with polyclonal rabbit anti-mouse vWF Ig (Abcam) following rinsing in PBS secondary biotinylated horse anti-rabbit Ig (Vector Labs). After another rinse in PBS, sections were incubated with Cy3-conjugated streptavidin (Jackson ImmunoResearch), and then mounted in glycerol-PBS. Images of the immunostained sections were acquired using the AxioObserver D2 inverted fluorescent microscope (Carl Zeiss) connected to a AxioCam HRm monochromatic digital camera, and stored as TIFF files. Fluorescence parameters were analysed automatically by Columbus software (Perkin Elmer).

### Assessment of nitrite in plasma and NOHb in erythrocytes

Blood samples were centrifuged (1000 x g, 5 min, 4 °C) to isolate plasma and erythrocytes. 50 μl plasma was mixed with 100 μl of cold ethanol and kept on ice for protein precipitation (30 min), centrifuged (14,000 x g, 5 min.) and the resulting supernatant was used immediately after sample preparation to determine nitrite concentration using reductive chemiluminescence analysis (Sievers* Nitric Oxide Analyzer NOA 280i), as described above.

EPR spectra of the isolated erythrocytes were used for the detection of nitrosylhemoglobin (NOHb), as described previously [14, 15]. Briefly, erythrocytes were snap-frozen in insulin syringes and EPR measurements were performed in in liquid nitrogen (77 K) using a Bruker EMX Plus spectrometer. Nitrosylhaemoglobin levels were expressed as the EPR amplitude of the second hyperfine line of the NOHb spectra in arbitrary units, and normalized to sample weight.

### Blood count and cytokine analysis in plasma

Blood count was performed immediately after blood collection using a blood counter (abc Vet, HORIBA). To obtain blood plasma, samples were centrifuged for 7 min (1000 x g). Acute-phase protein concentration in plasma was measured using magnetic-beads-based immunoassay (Multiplex, Millipore). SAA and IL-6 plasma concentrations were measured with ELISA kits (Invitrogen and R&D Systems, respectively).

### Statistical analysis

For statistical analysis, STATISTICA 10 software (StatSoft, Inc.) and OriginPro 9 software (OriginLab, Northampton, MA) were used. The Shapiro-Wilk test was used to verify whether the data were normally distributed. Levene’s test was used to determine homogeneity of variances. The significance of between-group differences was evaluated by the Kruskal-Wallis test or ANOVA with post-hoc Tukey multiple comparisons test or by Mann-Whitney U test, depending on the data distribution. Spearman or Pearson’s correlation coefficient test was used to assess dependence between two parameters. Results are presented as mean ± SEM. Differences between means were considered significant if *p* < 0.05.

## Results

### Primary tumour growth and lung metastases development

**Fig. 1:**
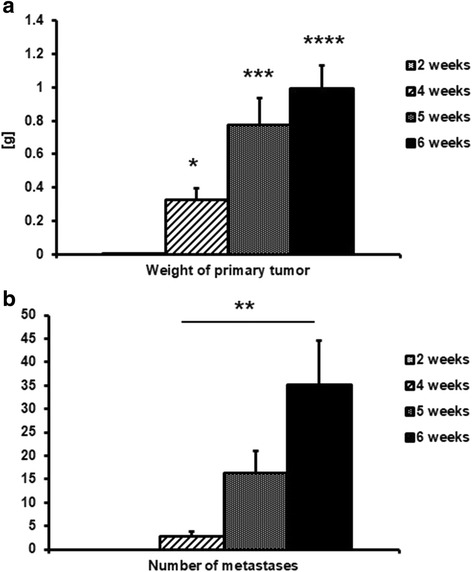
Weight of primary tumour (**a**), * indicates *p* < 0.05; *** indicates *p* < 0.001; **** indicates *p* < 0.0001 vs. 2-week group, number of metastases in the lungs (**b**), ** indicates *p* < 0.01 at 2, 4, 5 and 6 weeks after cancer cell inoculation in mice. Results are expressed as mean ± SEM, *n* = 10–17

Small palpable tumours (0.004 ± 0.001 g) were observed 2 weeks after cancer cell transplantation into mammary glands of BALB/c female mice. As shown in Fig. 1a, mean tumour weight increased progressively over the course of the study to a final weight of 0.99 ± 0.1 g, observed on the 6th week of the experiment.

No metastatic nodules were visible in lungs isolated from mice 2 weeks after cancer cell transplantation. However, the mean number of metastases developed in lungs isolated from tumour-bearing mice 4, 5 or 6 weeks after cancer cell transplantation gradually increased, reaching a mean of 35.2 ± 9.3 metastatic nodules at week 6 of the study (Fig. 1b).

### Early pro-inflammatory changes in the lungs and impaired NO production in the pre-metastatic phase

**Fig. 2:**
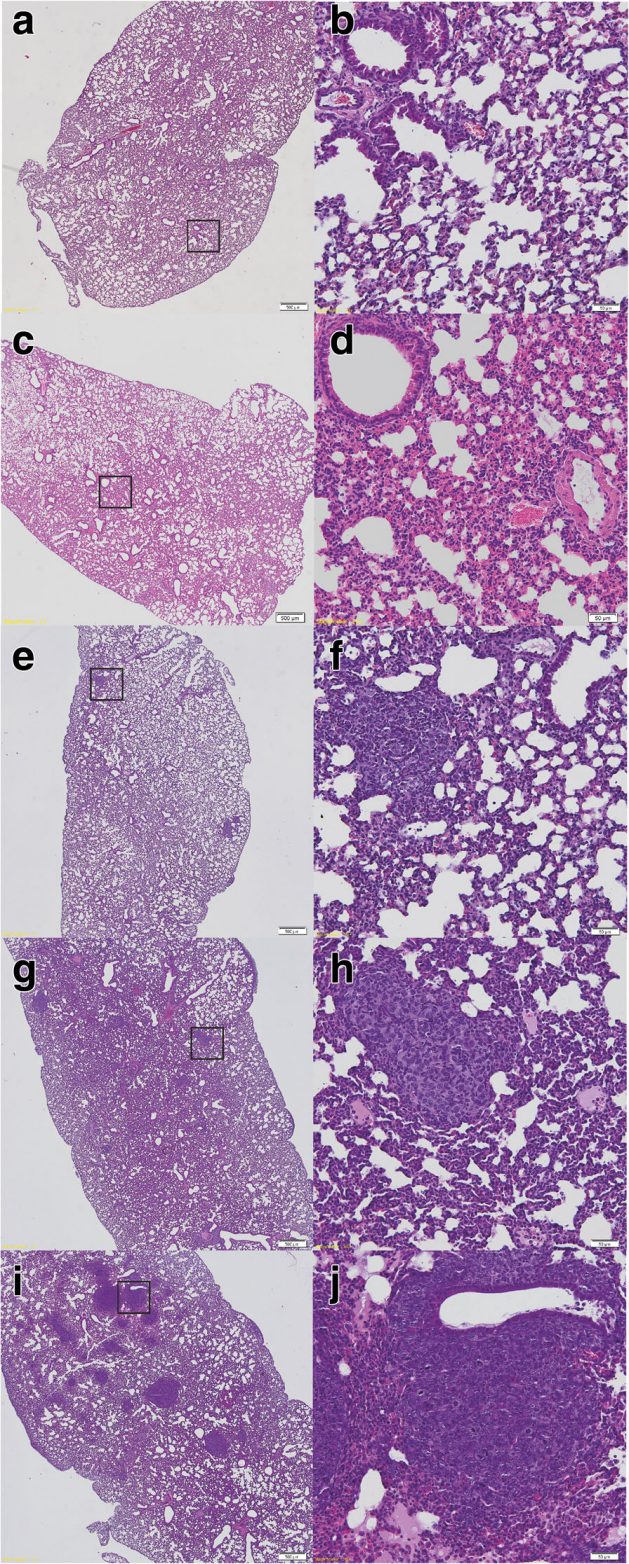
Histopathological analysis of the mice lung. Control – normal lung tissue (**a**, **b**); lungs at 2 weeks after cancer cell injection with visible sites of inflammation, mainly granulocyte infiltration (**c**, **d**); 4-week group lungs with few small metastatic focuses (**e**, **f**); 5-week group lungs with many visible scattered metastatic sites of different sizes (**g**, **h**); and lungs at 6 weeks after cancer cell injection, with numerous, extensive, merging metastatic areas (**i**, **j**). Magnification 20×: **a**, **c**, **e**, **g**, **i**; 200×: **b**, **d**, **f**, **h**, **j** (magnification of chosen area)

**Fig. 3:**
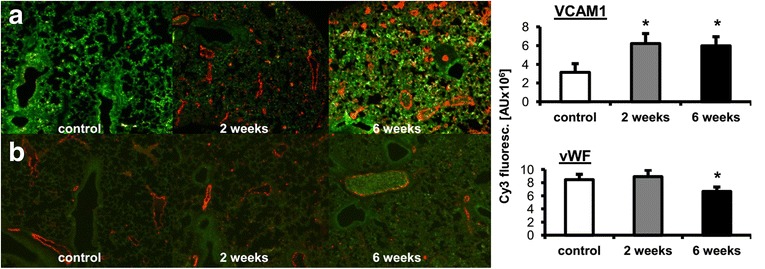
Results of VCAM-1 (**a**) and von Willebrant Factor (**b**) fluorescent immunostaining in lung tissue taken from mice 2 and 6 weeks after cancer cells inoculation. Statistically significant increase of VCAM-1 was observed in 2-week pre-metastatic group (*p* ≤ 0,05 vs control) as well as in 6-week metastatic group (p ≤ 0,05 vs control), whereas a slight decrease of vWF was present in 6-week metastatic group (*p* ≤ 0,05 vs control). Values are mean ± SEM, *n* = 5

As early as 2 weeks after cancer cell transplantation, local inflammatory cell infiltration into lung parenchyma was detected, identified as a population consisting mainly of granulocytes (Fig. 2d). However, no cancer cell clusters were observed in the lungs at this time point in microscopic cross-section preparations of the lungs (Fig. 2c). Inflammatory cells infiltration was accompanied by enhanced expression of VCAM-1 in the pulmonary vasculature, as evidenced by immunohistochemical staining (Fig. 3a). On the other hand, vWF staining in the lungs showed no significant difference between early pre-metastatic mice and controls (Fig. 3b). Moreover, nitrite and nitrate concentrations in buffer from isolated lungs perfused in a non-recirculated manner were lower in mice 2 weeks after cancer cell inoculation as compared to control mice (nitrite: 0.938 ± 0.301 μM and 1.749 ± 0.346 μM in tumour-bearing and control animals, respectively; nitrate: 3.00 ± 0.013 μM and 4.714 ± 0.445 μM in tumour-bearing and control animals, respectively; *n* = 3 in both groups).

### Lung histopathology in the advanced metastatic phase

In contrast to the lungs isolated from tumour-bearing mice at 2 weeks following cancer cell transplantation, lungs analysed at 4 weeks displayed small, clear-cut metastatic foci (Fig. 2e, f). 5 weeks after cancer cell transplantation, scattered metastatic sites of different sizes were observed in the lung parenchyma and under the pleura (Fig. 2g, h). In the most advanced stage of metastatic disease progression, 6 weeks after 4 T1 cell engraftment, numerous, extensive, merging metastatic foci were evident (Fig. 2i, j).

### Development of cancer-associated systemic inflammation

#### Leukocytosis and other changes in blood count

**Fig. 4:**
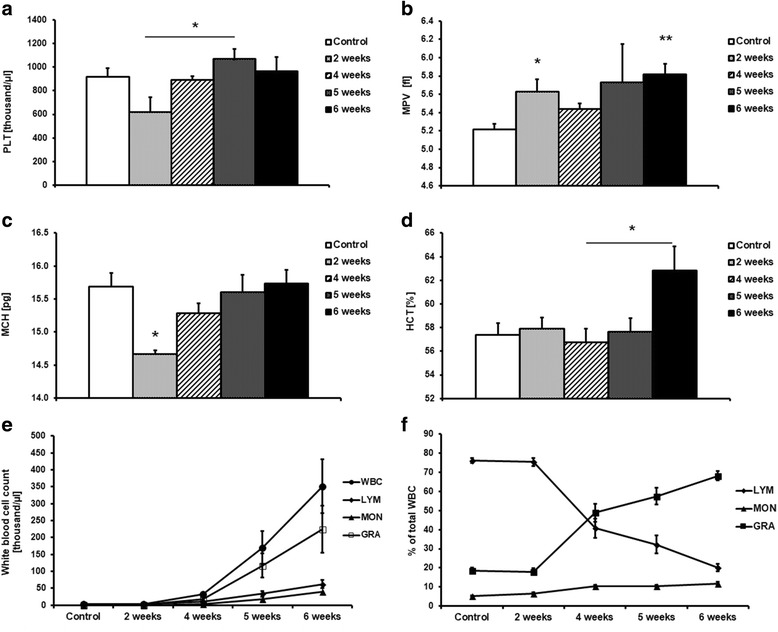
Blood counts in controls and mice at 2, 4, 5 and 6 weeks after cancer cell transplantation. Number of platelets (PLT) (**a**); number of red blood cells (RBC) (**b**); mean corpuscular haemoglobin (MCH) (**c**); haematocrit (HCT) (**d**); concentration of leukocytes (**e**); and percentage of monocytes [MON] lymphocytes [LYM] and granulocytes [GRA] in leukocytes fraction (**f**) in control mice and in mice at 2, 4, 5 and 6 weeks after cancer cell inoculation. Results are expressed as mean ± SEM, *n* = 6–12, * indicates *p* < 0.05

At 2 weeks after cancer cell transplantation, mild changes in the blood count of tumour-bearing mice were found, including a drop in platelet counts that normalized over the course of the study (Fig. 4a) and an increase in mean platelet volume (MPV) that remained at an elevated level until the late stage of metastatic disease development (Fig. 4b). A significant decrease in mean cell haemoglobin (MCH) was also noted at 2 weeks (Fig. 4c), while in the late stage of disease (6 weeks), haematocrit (HCT) and average red blood cell volume (MCV) were significantly increased (Fig. 4d; Table 1), compensating for the drop in MCH seen in the early stage of cancer progression (2 weeks).

**Table 1:** Blood counts in control mice and mice at 2, 4, 5 and 6 weeks after cancer cells inoculation

	Control	2nd week	4th week	5th week	6th week
PLT [K. μl^− 1^]	916.30 ± 47.39	618.75 ± 128.13#	892.81 ± 30.04	1067.25 ± 87.86	964.58 ± 119.81
MPV [fl]	5.21 ± 0.06	5.63 ± 0.13*	5.44 ± 0.06	5.73 ± 0.42	5.82 ± 0.12**
RBC [M. μl^−1^]	10.37 ± 0.17	10.50 ± 0.15	10.16 ± 0.22	10.27 ± 0.23	10.72 ± 0.30
MCH [pg]	15.69 ± 0.20	14.67 ± 0.06*	15.28 ± 0.15	15.60 ± 0.27	15.74 ± 0.21
HCT [%]	57.40 ± 0.96	57.92 ± 0.93	56.78 ± 1.12$	57.63 ± 1.15	62.84 ± 2.01
MCV [fl]	55.33 ± 0.18	55.15 ± 0.31	55.92 ± 0.32	55.10 ± 0.32	58.75 ± 0.70*
WBC [K. μl^−1^]	3.87 ± 0.21	2.94 ± 0.26	31.80 ± 6.14	169.35 ± 49.59**	351.50 ± 79.54****
LYM [K. μl^−1^]	2.88 ± 0.15	2.65 ± 0.60	9.96 ± 1.26	33.62 ± 8.15**	62.19 ± 11.43****
MON [K. μl^−1^]	0.15 ± 0.02	0.15 ± 0.02	3.59 ± 0.76	18.46 ± 5.67**	40.53 ± 8.15****
GRA [K. μl^−1^]	0.84 ± 0.08	0.62 ± 0.09	18.46 ± 4.54	117.27 ± 36.28*	224.36 ± 69.17****
LYM [%]	76.14 ± 1.39	75.35 ± 2.00	40.83 ± 5.03****	32.19 ± 4.69****	20.09 ± 1.86****
MON [%]	5.25 ± 0.26	6.69 ± 0.45	10.27 ± 0.83****	10.34 ± 0.64****	11.74 ± 0.98****
GRA [%]	18.61 ± 1.39	17.97 ± 1.71	48.91 ± 4.70*	57.47 ± 4.43***	68.17 ± 2.48****

Data are expressed as mean ± SEM

*PLT* platelets, *MPV* mean platelet volume, *RBC* red blood cells, *MCH* mean cell haemoglobin, *HCT* haematocrit, *MCV* mean corpuscular volume, *WBC* white blood cells, *LYM* lymphocytes, *MON* monocytes, *GRA* granulocytes

*p < 0.05, **p < 0.01, ***p < 0.001, and **** *P* < 0.0001 vs. control group; #*p* < 0.05 vs. 5-week group; $*p* < 0.05 vs. 6-week group, (*n* = 8–12)

The most pronounced alterations in the blood count profile were observed in the late stages of metastatic disease and involved profound and progressive leukocytosis observed at 4–6 weeks after cancer cell transplantation (Fig. 4e; Table 1). Although the absolute numbers of granulocytes [GRA], monocytes [MON], and lymphocytes [LYM] increased, the percentage changes indicated a substantial increase in granulocyte count, a mild increase in monocyte count, and a relative decrease in lymphocyte count (Fig. 4f). In fact, in control animals as well as in the early stage of disease progression in tumour-bearing mice (2 weeks), the most abundant WBCs were lymphocytes, while in the advanced stages of cancer progression (4–6 weeks) the most abundant WBCs were granulocytes (Fig. 4f; Table 1).

There was a high correlation between number of metastases and leukocytes, irrespective of whether the change in total white blood cells, or in granulocytes, lymphocytes, and monocytes, were analysed (Table 2).

**Table 2:** The correlation between the number of metastases in the lungs of tumour-bearing mice and blood parameters assessed via the Spearman correlation coefficient test

The pair of parameters	R Spearman	p
Number of metastases & WBC	0.795611	0.000000
Number of metastases & LYM	0.785315	0.000000
Number of metastases & MON	0.788080	0.000000
Number of metastases & GRA	0.755175	0.000001
Number of metastases & MCV	0.632756	0.000102
Number of metastases & MCH	0.515611	0.002525

#### Changes in plasma concentration of acute phase proteins and IL-6

**Fig. 5:**
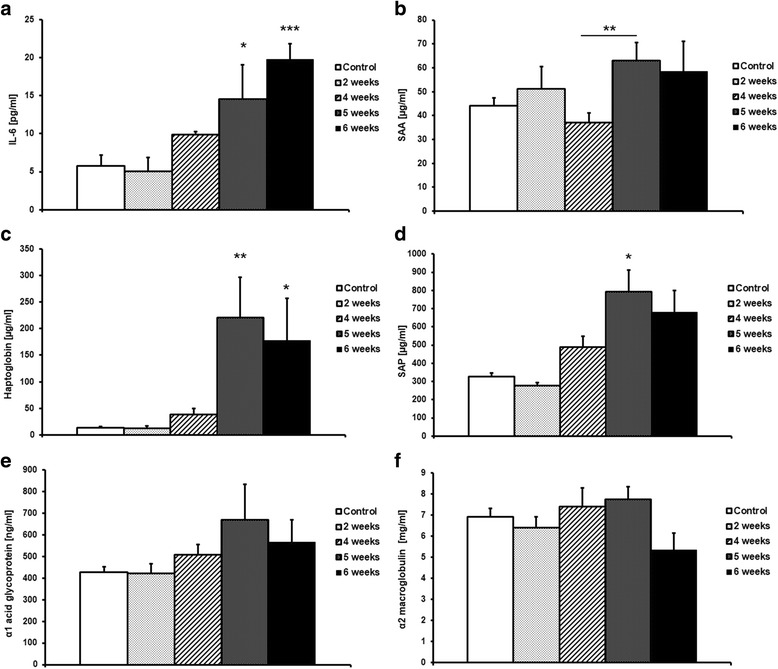
Concentration of IL-6 (**a**) in plasma from controls and mice at 2, 4, 5 and 6 weeks after cancer cell transplantation. Results are expressed as mean ± SEM, *n* = 6–14, * indicates *p* < 0.05; ** indicates *p* < 0.01 vs. control. Changes in concentration of acute phase proteins: Serum amyloid A (**b**); Haptoglobin (**c**); Serum amyloid protein (**d**); α1 acid glycoprotein (**e**) and α2 macroglobulin (**f**) in plasma from control mice and mice at 2, 4, 5 and 6 weeks after cancer cell transplantation. Results are expressed as mean ± SEM, *n* = 7–16, ** indicates *p* < 0.01 vs. control

Along with tumour progression, there was a gradual increase in plasma IL-6 concentration in tumour-bearing mice. In the early stage, 2 weeks and 4 weeks after cancer cell transplantation, plasma IL-6 concentration was comparable to that of control mice, while at 5 and 6 weeks after cancer cell transplantation, there was a significant, 3- to 4-fold elevation in plasma IL-6 concentration compared to control (14.5 ± 4.5 pg/ml and 19.8 ± 2 pg/ml vs. 5.8 ± 1.4 pg/ml, respectively; Fig. 5a). Quite a similar response pattern was observed for acute phase proteins in plasma. The concentrations of serum amyloid A (SAA), haptoglobin and serum amyloid protein (SAP) (Fig. 5b-d) were significantly increased in tumour-bearing mice at 5 and 6 weeks following cancer cell inoculation. Plasma levels of haptoglobin, IL-6 and SAA correlated with the number of lung metastases in tumour-bearing mice (Table 3). On the other hand, metastases development did not significantly influence plasma concentrations of α1 acid glycoprotein (Fig. 5e) or α2 macroglobulin (Fig. 5f).

**Table 3:** The correlation between number of metastases in the lungs of tumour-bearing mice and Interleukin-6 (IL-6), Serum Amyloid A (SAA) and haptoglobin, as assessed via the Spearman correlation coefficient test

The pair of parameters	R Spearman	p
Number of metastases & IL-6	0.655558	0.000014
Number of metastases & SAA	0.554662	0.000447
Number of metastases & Haptoglobin	0.814386	0.013844

### Changes in NO-dependent function in aorta

**Fig. 6:**
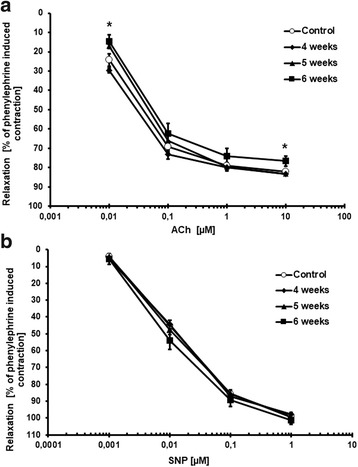
Endothelium-dependent vasorelaxation induced by Ach (**a**) and endothelium-independent vasorelaxation induced by SNP (**b**) in aortic rings precontracted with phenylephrine; * indicates *p* < 0.05 for group 6 weeks after cancer cell transplantation vs. control group. Results are expressed as mean ± SEM, *n* = 6–13 (2–4 rings from one mouse)

In all experimental groups, acetylcholine (Ach) induced a dose-dependent vasodilation of phenylephrine-precontracted aortic rings that was entirely inhibited by L-NAME. The Ach-induced response in aorta was not changed at 2, 4 and 5 weeks after cancer cell inoculation, but was impaired in the latest stage of metastatic cancer progression (6 weeks) as compared to control mice (Fig. 6a). In contrast, the endothelium-independent response induced by SNP remained unaffected for all experimental groups at all time points (Fig. 6b).

**Fig. 7:**
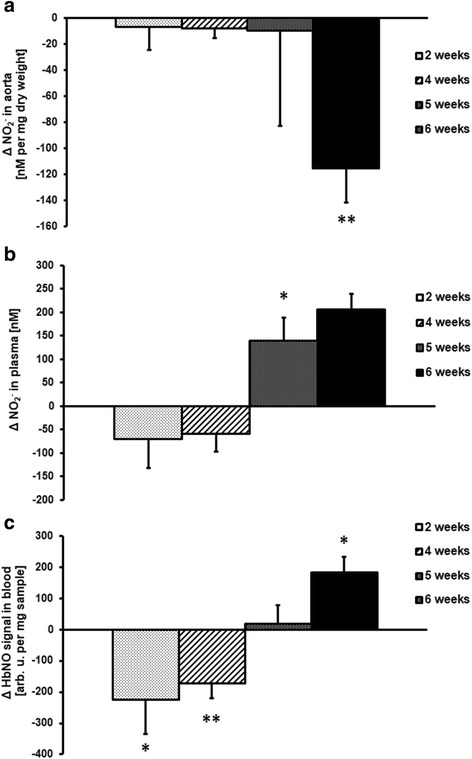
Basal nitrite production in incubated aorta rings (**a**), plasma nitrite concentration (**b**) and nitrosylhemoglobin level (**c**) in mice at 2, 4, 5 and 6 weeks after cancer cell transplantation (Results presented as Δ metastasis – control, showing [%] difference from appropriate control groups at corresponding age (2, 4, 5 and 6 weeks after cancer cell inoculation). * indicates *p* < 0.05 and ** indicates *p* < 0.01. Results are expressed as mean ± SEM, *n* = 3 (control groups) and *n* = 6 (metastasis groups)

Parallel to the impairment of the NO-dependent Ach-induced response, a deterioration of nitrite production by the endothelium was observed in the isolated aortic rings. As shown in Fig. 7a, basal nitrite production was significantly lower in aortas isolated from tumour-bearing mice at 6 weeks after cancer cell inoculation, supporting an impairment of NO-dependent function.

### Changes in systemic NO bioavailability

Mean plasma nitrite concentration in control mice was 487 ± 45 nM. Interestingly, in the early stage of cancer development (2 and 4 weeks after cancer cell transplantation) plasma nitrite concentration and NOHb concentration were lower (significant for NOHb, *p* < 0.05) compared to control mice (Fig. 7b, c).

In contrast to the lower concentration of NO_x_ metabolites in the early phase of metastasis, the concentrations of both NOHb and nitrite were significantly increased at 6 weeks after cancer cell transplantation (Figs. 6c and 7b).

### Changes in PGI_2_-dependent function in the aorta

**Fig. 8:**
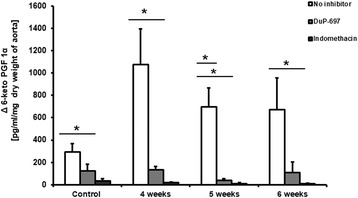
Changes in 6-keto PGF_1α_ concentration in effluents from aortic rings during 27 min of incubation in Krebs buffer with no inhibitor and in the presence of COX-1 selective inhibitor DuP-697 (1 μM) or nonselective COX-1 and COX-2 inhibitor indomethacin (5 μM). Results are expressed as mean ± SEM, *n* = 5–6, * indicates *p* < 0.05; ** indicates *p* < 0.01

Impairment of NO-dependent function in the aorta was proceeded by persistent compensatory up-regulation of PGI_2_ production, as shown through an increased concentration of the stable PGI_2_ metabolite 6-keto-PGF1_α_ in the effluent of aortic rings isolated from tumour-bearing mice at 4–6 weeks following cancer cell inoculation. Both DuP-697 (1 μM) and indomethacin (5 μM) profoundly inhibited PGI_2_ release from aortic rings, suggesting that COX-2 was the main contributor to the upregulated PGI_2_ production in the vascular wall (Fig. 8).

### Changes in vWF expression in aorta

**Fig. 9:**
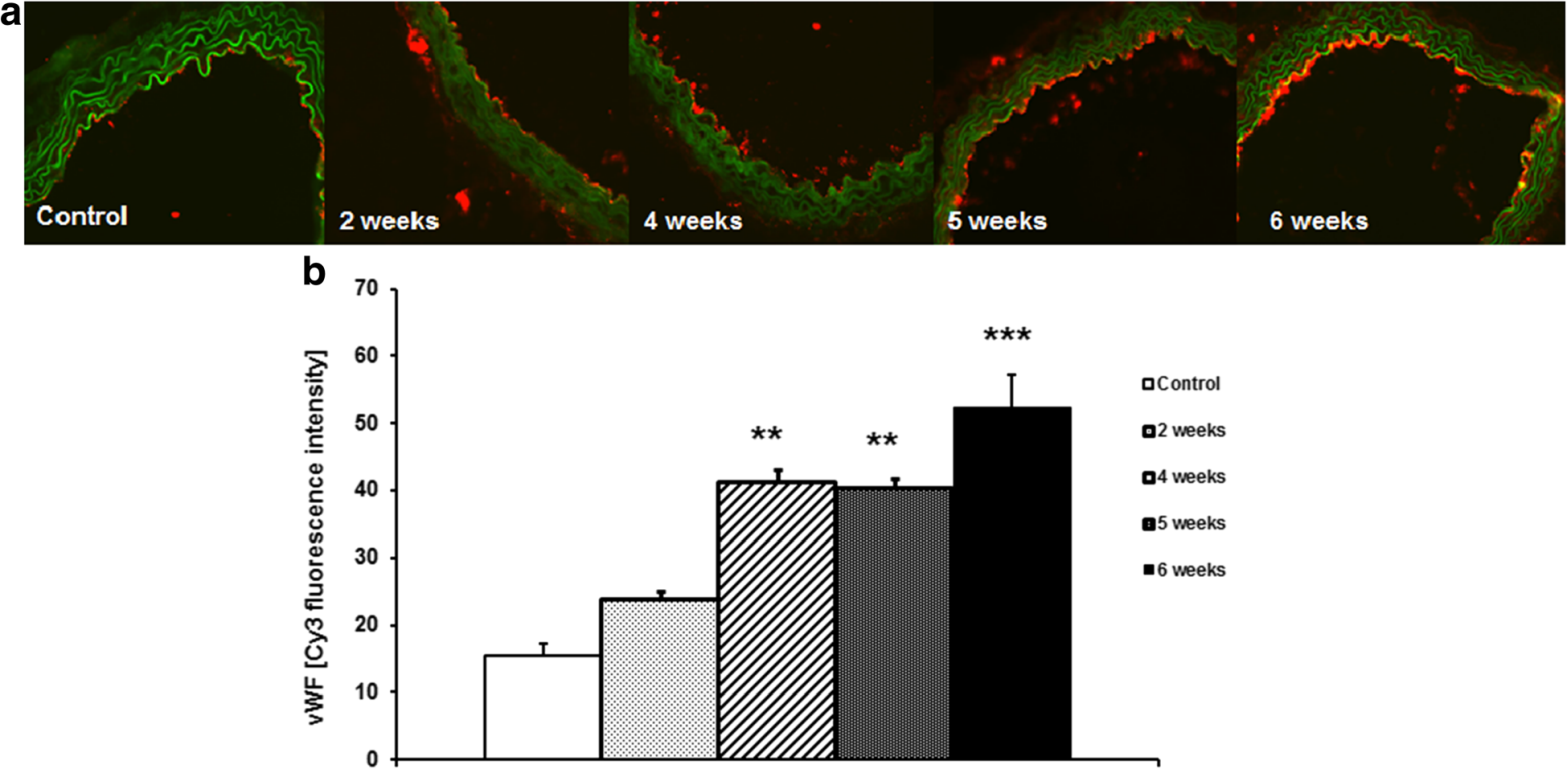
Representative microphotographs of endothelial von Willebrand Factor (vWF) expression in cross-sectional slides of the thoracic aorta (**a**) and summarized results (**b**) analysed by Columbus texture analysis of vWF Cy3 fluorescence in aortic rings from control mice and mice at 2, 4, 5 and 6 weeks after cancer cell inoculation. ** indicates *p* < 0.01; *** indicates *p* < 0.001. Results are expressed as mean ± SEM, *n* = 4

As shown in Fig. 9, in the aortic endothelium of tumour-bearing mice, vWF expression was increased compared to control animals. Up-regulated expression of vWF was observed as early as 2 weeks after cancer cell inoculation and progressively increased through the later stages of metastases development (Fig. 9a, b).

## Discussion

Healthy endothelium inhibits formation of metastasis, while dysfunctional endothelium has an opposite effect [1, 7]. On the other hand, systemic endothelial dysfunction developing along with tumour progression and metastasis contributes to cardiovascular complications in cancer patients [6]. The present work has taken advantage of the prolonged time course of the disease progression in the spontaneously metastasizing, orthotopic, 4 T1 murine breast cancer model in order the characterize the temporal relationships between the development of local, pulmonary, and systemic endothelial dysfunction in relation to primary tumour growth and lung metastases formation. We showed that an early decrease in NO bioavailability was temporarily associated with the development of early metastasis and lung inflammation, while late systemic impairment of NO-dependent function in the aorta coincided with robust, systemic, cancer-related inflammation. Our results show that the local pulmonary endothelial response to murine metastatic breast cancer development involves pulmonary inflammation, granulocyte infiltration and decreased NO bioavailability in the lung. On the other hand, impairment of NO-dependent function in the aorta occurred only in the late phase of metastasis formation, most likely as a consequence of robust, cancer-related systemic inflammation mediated by IL-6, TNFα [16] or other mechanisms [17]. Interestingly, impairment of NO-dependent function was associated with the upregulation of COX-2-derived PGI_2_ production, which could represent a compensatory mechanism. Indeed, PGI_2_, the most potent endogenous anti-platelet agent, has been shown to prevent cancer metastasis [11, 12]. The same activity has also been reported for PGI_2_ analogues [16, 17] and PGI_2_-releasing compounds [20].

Although it was recently reported that the development of metastatic breast carcinoma in mice is associated with changes in vascular reactivity of the aorta [16], to our knowledge the present work reports for the first time the temporal relationships between the development of the local, pulmonary endothelial dysfunction that is a pre-requisite for development of metastasis in the lung and the systemic endothelial dysfunction and vascular inflammation that affect general cardiovascular health.

Taking advantage of the slowly progressing 4 T1 breast cancer-based model established by our group [14, 17], which relies on transplantation of a low number of 4 T1 cancer cells and is similar to the recently-reported optimized 4 T1 triple negative syngeneic micro- and macro-metastasis model by Bailey-Downs [21], we clearly differentiated between the occurrence of early endothelial dysfunction in lungs at the pre-metastatic/early metastatic stage and development of peripheral endothelial dysfunction in the conduit artery at the late metastastic stage. These results suggest different mechanisms involved in these responses. The early pre-metastatic phase was characterized by clear-cut evidence of pulmonary inflammation and granulocytic cellular infiltration. The presence of leukocyte infiltration is consistent with previous work [22–24] demonstrating that 4 T1 tumour-bearing mice display granulocytic infiltrates in metastatic sites [25] and the result supports the concept of a pre-metastatic niche formed in the pulmonary target tissue before it is invaded by migrating cancer cells [26]. This hypothesis is also supported by the VCAM-1 immunofluorescent staining results showing a pro-inflammatory phenotype in the pulmonary endothelium as early as 2 weeks following cancer cell transplantation, at the early stage of the metastatic process.

Based on the analysis in the isolated perfused lung preparation, we found that nitric oxide production was decreased in the early phase of metastasis development. Impairment of NO bioavailability may contribute to the adhesion of cancer cells and neutrophils to the endothelium and may facilitate cancer cells extravasation [25–28]. Given that NO displays anti-platelet activity and that platelets are involved in arresting tumour cells in the vasculature at metastatic extravasation sites [29–32], a low-NO microenvironment could also affect the platelet-cancer cell interaction, facilitating metastasis. Interestingly, we detected a decrease in platelet number in the early phase of metastasis, which could reflect an increased platelet-cancer cell interaction. Altogether, our findings of early NO deficiency seem to be important in the context of pathogenic endothelial-dependent mechanisms of early metastasis. NO-deficient pre-metastatic niche formation may promote early metastasis in the lung involving cancer cells, leucocytes and platelets.

Interestingly, the pre-metastatic/early metastatic phase was associated with a transient decrease in systemic NO availability, as evidenced by a drop in NOHb content, as well as decreased nitrite concentration in plasma. These results suggest that the magnitude of the impaired NO production in the lung was high enough to impose an effect on systemic NO bioavailability. Given that the pulmonary endothelial surface represents approximately 30% of the total endothelial surface of the cardiovascular system, this finding is not surprising. Later in the course of metastatic development, nitrite and NOHb increased, an effect most likely linked to systemic inflammation and NOS-2 induction [33].

In contrast to the pre-metastatic and early metastatic phases, the late metastatic phase of disease (5–6 weeks after cancer cell transplantation) was characterized by a robust systemic inflammation, including substantial leucocytosis, increases in RBC number, MCV and HCT, elevated platelet number, mean platelet volume, as well as increased plasma concentration of acute phase proteins (haptoglobin, serum amyloid A) and IL-6. Leukocytosis seems to be compatible with the production of colony-stimulating factors released by tumour cells in 4 T1 tumour-bearing mice, enhanced myelopoiesis in bone marrow and extramedullary myelopoiesis occurring in the spleen [25], and may support metastasis by creating an environment that facilitates the recruitment and growth of circulating tumour cells. Indeed, G-CSF neutralization was shown to diminish tumour cell infiltration into lungs, and consequently to reduce metastasis [24].

In our study, progression of metastasis was also associated with an increased concentration of IL-6, a major pro-inflammatory cytokine that affects cell proliferation, survival, and metabolism and is known to be associated with the progression of various types of cancer [34–36]. IL-6 stimulates the expression of adhesion molecules on the endothelial surface that could favour the adhesion of tumour cells [37]. Interestingly, since IL-6 has been found to be elevated in cancer patients with distal metastases, as compared with non-metastatic patients, an increased IL-6 concentration has been proposed as a prognostic marker in some types of metastatic cancer [37, 38]. In the present work, metastasis was linked with the activation of the acute phase response, as evidenced by the elevation of serum amyloid A, serum amyloid P and haptoglobin, a response that could be triggered by an increased IL-6 concentration. It has previously been reported that haptoglobin is elevated in breast cancer and may be indicative of metastases [40, 41]. Interestingly, altered glycan content in some acute phase proteins, for example, haptoglobin, interact with a number of receptors on macrophages in the tumour microenvironment, potentially modulating macrophage activity and thereby contributing to tumour cell survival, growth, and metastasis [41, 42]. In turn, SAA stimulates its own transcription as well as that of the proinflammatory S100A8 and S100A9 proteins, strongly enhancing adhesion, migration and invasion of human and mouse tumour cells [43]. Thus, the acute phase response may contribute to the metastasis formation via various mechanisms [41]. In our study, metastasis in 4 T1 tumour-bearing mice was associated with substantial leukocytosis, activation of IL-6-dependent mechanisms and an acute phase response, all of which could contribute to the further acceleration of metastasis formation [23, 39, 40]. In fact, there was a high correlation between leukocyte number, IL-6, haptoglobin, SAA plasma concentration and a number of lung metastases in tumour-bearing mice. At the stage of advanced metastasis associated with robust systemic inflammation, the NO-dependent response in aorta was impaired, while the endothelium-independent response was preserved. Interestingly, downregulation of a NO-dependent endothelial activity was associated with a compensatory increase in COX-2-mediated synthesis of PGI_2_, a vasoprotective molecule with known anti-metastatic potential [11, 12, 18–20, 44].

In our hands, impaired NO-dependent function in the aorta was associated with up-regulation of vWF expression, suggesting a pro-thrombotic phenotype of dysfunctional endothelium in aorta, compatible with cancer-related activation of pro-thrombotic endothelial mechanisms [6]. Interestingly, vWF did not increase in the pulmonary endothelium, but was even lower in the late stage of metastasis formation compatible with a possible involvement of vWF in cancer cell metastasis to the lungs [45, 46].

## Conclusion

In the present work in orthotopic murine model of metastatic 4 T1 breast cancer we have characterized the temporal relationships between the development of endothelial dysfunction in the pulmonary and systemic circulation in relation to primary tumour growth and lung metastases. We have demonstrated that the local pulmonary endothelium response to cancer cells, that is the pre-requisite response for the development of metastases in the lung, involves decreased NO bioavailability in the lung, that is also reflected by a decrease in systemic NO bioavailability and is associated with pulmonary inflammation with granulocyte infiltration. All these early changes precedes the systemic inflammation and systemic endothelial dysfunction induced by cancer development. Late impairment of endothelial NO-dependent function in the aorta was associated with compensatory upregulation of the COX-2-derived PGI_2_ pathway and coincided with robust, systemic, cancer-related inflammation. Our results obtained in an murine, orthotopic model of breast cancer metastasis, relevant to human pathology, suggest a heterogeneity of mechanisms of the early endothelial dysfunction in metastatic organ paving the way for metastases formation and the late systemic endothelial dysfunction contributing to systemic vascular inflammation.

## Abbreviations

Ach: Acetylcholine
COX-2: Cyclooxygenase-2
CTCs: Circulating tumour cells
DMSO: Dimethyl sulfoxide
EMT: Epithelial mesenchymal-like-transition
HPLC: High-pressure liquid chromatography
IL-6: Interleukin 6
K-H buffer: Krebs-Hanseleit buffer
L-NAME: N-*ω*-Nitro -L-Arginine Methyl Ester
NO: Nitric oxide
NOHb: Nitrosylhaemoglobin
NOS: Nitric oxide synthase
PDGF: Platelet-derived growth factor
PGI_2_: Prostacyclin
Phe: Phenylephrine
SAA: Serum amyloid A
SNP: Sodium nitroprusside
TGF-β: Transforming growth factor β
VCAM-1: Vascular cell adhesion molecule 1
VEGF: Vascular endothelial growth factor
vWF: von Willebrand factor

## References

[1] Widlansky ME, Gokce N, Keaney JF, Vita JA (2003). The clinical implications of endothelial dysfunction. J Am Coll Cardiol.

[2] Matsuzawa Y, Guddeti RR, Kwon T, Lerman LO, Lerman A (2014). Treating coronary disease and the impact of endothelial dysfunction. Prog Cardiovasc Dis.

[3] Walczak M, Suraj J, Kus K, Kij A, Zakrzewska ACS (2015). Towards a comprehensive endothelial biomarkers profiling and endothelium-guided pharmacotherapy. Pharmacol Rep.

[4] Daher IN, Daigle TR, Bhatia N, Durand J-B (2012). The prevention of cardiovascular disease in cancer survivors. Tex Heart Inst J.

[5] Shenoy C, Klem I, Crowley A (2011). Cardiovascular complications of breast cancer therapy in older adults. Oncologist.

[6] Morganti M, Carpi A, Nicolini A, Gorini I, Glaviano B, Fini M (2002). Atherosclerosis and cancer: common pathways on the vascular endothelium. Biomed Pharmacother.

[7] Reymond N, d’Água BB, Ridley AJ (2013). Crossing the endothelial barrier during metastasis. Nat Rev Cancer.

[8] Roblek M, Calin M, Schlesinger M, Stan D, Zeisig R, Simionescu M (2015). Targeted delivery of CCR2 antagonist to activated pulmonary endothelium prevents metastasis. J Control Release.

[9] Schlesinger MBG (2015). Vascular cell adhesion molecule-1 (VCAM-1)--an increasing insight into its role in tumorigenicity and metastasis. Int J Cancer.

[10] Franses JW, Drosu NC, Gibson WJ, Chitalia VC, Edelman ER (2013). Dysfunctional endothelial cells directly stimulate cancer inflammation and metastasis. Int J Cancer.

[11] Honn K, Cicone B, Skoff A (1981). Prostacyclin: a potent antimetastatic agent. Science (80- ).

[12] Schneider MR, Tang DG, Schirner M, Honn KV (1994). Prostacyclin and its analogues: antimetastatic effects and mechanisms of action. Cancer Metastasis Rev.

[13] Wojcik T, Buczek E, Majzner K, Kolodziejczyk A, Miszczyk J, Kaczara P (2015). Comparative endothelial profiling of doxorubicin and daunorubicin in cultured endothelial cells. Toxicol in Vitro.

[14] Smeda M, Kieronska A, Proniewski B, Jasztal A, Selmi A, Wandzel K (2018). Dual antiplatelet therapy with clopidogrel and aspirin increases mortality in 4T1 metastatic breast cancer-bearing mice by inducing vascular mimicry in primary tumour. Oncotarget.

[15] Kramkowski K, Leszczynska A, Przyborowski K, Kaminski T, Rykaczewska U, Sitek B, et al. Role of xanthine oxidoreductase in the anti-thrombotic effects of nitrite in rats *in vivo*. Platelets. 2016;27:245–53. [cited 2018 Apr 13] Available from: https://www.ncbi.nlm.nih.gov/pubmed/26374946. 10.3109/09537104.2015.1083545 26374946

[16] Dalaklioglu S, Tasatargil A, Kale S, Tanriover G, Dilmac S, Erin N (2013). Metastatic breast carcinoma induces vascular endothelial dysfunction in Balb-c mice: role of the tumor necrosis factor-α and NADPH oxidase. Vasc Pharmacol.

[17] Cedervall J, Dimberg A, Olsson AK. “Tumor-Induced Local and Systemic Impact on Blood Vessel Function,” Mediat Inflamm. 2015:1–8. Article ID 418290. 10.1155/2015/418290. 10.1155/2015/418290 PMC4685129 26770016

[18] Tzanakakis GN, Agarwal KCVM (1990). Inhibition of hepatic metastasis from a human pancreatic adenocarcinoma (RWP-2) in the nude mouse by prostacyclin, forskolin, and ketoconazole. Cancer.

[19] Sava G, Perissin L, Zorzet S, Piccini P, Giraldi T (1989). Antimetastatic action of the prostacyclin analog iloprost in the mouse. Clin Exp Metastasis.

[20] Blazejczyk A, Switalska M, Chlopicki S, Marcinek A, Gebicki J, Nowak M, Nasulewicz-Goldeman AWJ (2016). 1-methylnicotinamide and its structural analog 1,4-dimethylpyridine for the prevention of cancer metastasis. J Exp Clin Cancer Res.

[21] Bailey-Downs LC, Thorpe JE, Disch BC, Bastian A, Hauser PJ, Farasyn T, et al. Development and characterization of a preclinical model of breast cancer lung micrometastatic to macrometastatic progression. PLoS One. 2014;9 [cited 2014 Jun 16] Available from: http://www.ncbi.nlm.nih.gov/pmc/articles/PMC4039511/10.1371/journal.pone.0098624 PMC4039511 24878664

[22] Wculek SK, Malanchi I (2015). Neutrophils support lung colonization of metastasis-initiating breast cancer cells. Nature.

[23] Tabariès S, Ouellet V, Hsu BE, Annis MG, Rose AA, Meunier L (2015). Granulocytic immune infiltrates are essential for the efficient formation of breast cancer liver metastases. Breast Cancer Res.

[24] Kowanetz M, Wu X, Lee J, Tan M, Hagenbeek T, Qu X (2010). Granulocyte-colony stimulating factor promotes lung metastasis through mobilization of Ly6G+Ly6C+ granulocytes. Proc Natl Acad Sci U S A.

[25] DuPre’ S a, Hunter KW (2007). Murine mammary carcinoma 4T1 induces a leukemoid reaction with splenomegaly: association with tumor-derived growth factors. Exp Mol Pathol.

[26] Sleeman JP (2012). The metastatic niche and stromal progression. Cancer Metastasis Rev.

[27] Chello M, Mastroroberto P, Perticone F, Celi VCA (1998). Nitric oxide modulation of neutrophil-endothelium interaction: difference between arterial and venous coronary bypass grafts. J Am Coll Cardiol.

[28] Kubes P, Suzuki M, Granger DN (1991). Nitric oxide: an endogenous modulator of leukocyte adhesion. Proc Natl Acad Sci U S A.

[29] Bambace NM, Holmes CE (2011). The platelet contribution to cancer progression. J Thromb Haemost.

[30] Erpenbeck L, Scho MP (2010). Review article deadly allies : the fatal interplay between platelets and metastasizing cancer cells. Blood.

[31] Gay L, Felding-Habermann B (2011). Contribution of platelets to tumour metastasis. Nat Rev Cancer.

[32] Lu Y, Yu T, Liang H, Wang J, Xie J, Shao J (2014). Nitric oxide inhibits hetero-adhesion of cancer cells to endothelial cells: restraining circulating tumor cells from initiating metastatic cascade. Sci Rep.

[33] Lechner M, Lirk P, Rieder J (2005). Inducible nitric oxide synthase (iNOS) in tumor biology: the two sides of the same coin. Semin Cancer Biol.

[34] Sansone P, Storci G (2007). IL-6 triggers malignant features in mammospheres from human ductal breast carcinoma and normal mammary gland. J Clin Invest.

[35] Schafer Z, Brugge J (2007). IL-6 involvement in epithelial cancers. J Clin Invest.

[36] Utsumi K, Takai Y, Tada T, Ohzeki S, Fujiwara H, Hamaoka T (1990). Enhanced production of IL-6 in tumor-bearing mice and determination of cells responsible for its augmented production. J Immunol.

[37] Brodt P, Fallavollita L, Bresalier RS, Meterissian S, Norton CR, Wolitzky B a (1997). Liver endothelial E-selectin mediates carcinoma cell adhesion and promotes liver metastasis. Int J Cancer.

[38] Tawara K, Oxford JT, Jorcyk CL (2011). Clinical significance of interleukin (IL)-6 in cancer metastasis to bone: potential of anti-IL-6 therapies. Cancer Manag Res.

[39] Salgado R, Junius S, Benoy I, Van Dam P, Vermeulen P, Van Marck E (2003). Circulating interleukin-6 predicts survival in patients with metastatic breast cancer. Int J Cancer.

[40] Thompson DK, Haddow JE, Smith DE, Ritchie RF (1983). Elevated serum acute phase protein levels as predictors of disseminated breast cancer. Cancer.

[41] Dempsey E, Rudd PM (2012). Acute phase glycoproteins: bystanders or participants in carcinogenesis?. Ann N Y Acad Sci.

[42] Carlsson MC, Cederfur C, Schaar V, Balog CIA, Lepur A, Touret F (2011). Galectin-1-binding glycoforms of haptoglobin with altered intracellular trafficking, and increase in metastatic breast cancer patients. PLoS One.

[43] Hansen MT, Forst B, Cremers N, Quagliata L, Ambartsumian N, Grum-Schwensen B, et al. A link between inflammation and metastasis: serum amyloid A1 and A3 induce metastasis, and are targets of metastasis-inducing S100A4. Oncogene. 2014; Available from: http://www.ncbi.nlm.nih.gov/pubmed/24469032 10.1038/onc.2013.568 24469032

[44] Bar A, Olkowicz M, Tyrankiewicz U, Kus E, Jasinski K, Smolenski RT (2017). Functional and biochemical endothelial profiling in vivo in a murine model of endothelial dysfunction; comparison of effects of 1-methylnicotinamide and angiotensin-converting enzyme inhibitor. Front Pharmacol.

[45] Luo G-P, Ni B, Yang X, Wu Y-Z (2012). von Willebrand factor: more than a regulator of hemostasis and thrombosis. Acta Haematol.

[46] Terraube V, Pendu R, Baruch D, Gebbink MFBG, Meyer D, Lenting PJ (2006). Increased metastatic potential of tumor cells in von Willebrand factor-deficient mice. J Thromb Haemost.

